# Newborn weight nomograms in selected low and middle-income countries

**DOI:** 10.1038/s41598-023-39773-4

**Published:** 2023-08-29

**Authors:** Amy Sarah Ginsburg, Fyezah Jehan, Shabina Ariff, Muhammad Imran Nisar, Eric Schaefer, Valerie Flaherman, Akina Shrestha, Srijana Dongol, Victoria Laleau, Augusto Braima de Sa, Raimundo Co, Victoria Nankabirwa

**Affiliations:** 1 University of Washington, Seattle, WA USA; 2 Aga Khan University, Karachi, Pakistan; 3 Penn State College of Medicine, Hershey, PA USA; 4 University of California, San Francisco, 3333 California St., Box 0503, San Francisco, CA 94118 USA; 5 Kathmandu University School of Medical Sciences, Dhulikhel, Nepal; 6 International Partnership for Human Development, Bissau, Guinea-Bissau; 7 Makerere University, Kampala, Uganda

**Keywords:** Paediatrics, Neonatology, Paediatric research

## Abstract

Growth impairment is common in low- and middle-income countries (LMIC) and may begin during early infancy, increasing morbidity and mortality. To ensure healthy infant growth, healthcare providers in high-income countries (HIC) track newborn weight change using tools developed and validated in HIC. To understand the utility of these tools for LMIC, we conducted a secondary analysis to compare weight trajectories in the first 5 days of life among newborns born in our LMIC cohort to an existing HIC newborn weight tool designed to track early weight change. Between April 2019 and March 2020, a convenience sample of 741 singleton healthy breastfeeding newborns who weighed ≥ 2000 g at birth were enrolled at selected health facilities in Guinea-Bissau, Nepal, Pakistan, and Uganda. Using a standardized protocol, newborn weights were obtained within 6 h of birth and at 1, 2, 3, 4, and 5 days, and nomograms depicting newborn weight change were generated. The trajectories of early newborn weight change in our cohort were largely similar to published norms derived from HIC infants, with the exceptions that initial newborn weight loss in Guinea-Bissau was more pronounced than HIC norms and newborn weight gain following weight nadir was more pronounced in Guinea-Bissau, Pakistan, and Uganda than HIC norms. These data demonstrate that HIC newborn weight change tools may have utility in LMIC settings.

## Introduction

Data from high-income countries (HIC) show that newborns typically lose weight in the first few days after birth and then generally begin steady weight gain^1–3^. To ensure initiation of healthy newborn growth, changes in newborn weight are tracked by healthcare providers in HIC using existing tools specifically designed for the assessment of weight change in the first days after birth^4,5^. Such tracking also might be beneficial in low- and middle-income countries (LMIC) where newborn growth impairment is common and increases the risk of morbidity and mortality, because early identification of growth impairment may allow prompt clinical intervention such as lactation or thermal support to prevent adverse outcomes.

The applicability of existing HIC tools for the tracking of initial weight change among LMIC newborns has not yet been reported. In the absence of a validated weight change tool, healthcare providers may instead use World Health Organization (WHO) Child Growth Standards (CGS) charts or for preterm infants may use International Fetal and Newborn Growth Consortium for the 21st Century (INTERGROWTH-21st) charts. However, WHO CGS charts and INTERGROWTH-21st charts were designed to assess weight for age in the context of population norms, rather than change in weight for an individual infant over time, and so may be unable to detect excessive weight loss for infants born with normal or above average birth weight. This has important implications for management because excessive weight loss in the days following birth may indicate physiologic concerns even when absolute weight remains normal for age.

One tool commonly used in HIC to monitor initial newborn weight change is the Newborn Weight Tool (NEWT), which was developed using data from 108,907 healthy exclusively breastfed newborns ≥ 36 weeks’ gestation in the United States^6^. Our team previously reported that we collected daily weights over the first 5 days among newborns ≥ 2000 g in selected LMIC to describe newborn weight change and predictors of being underweight^7^. To better understand how initial newborn weight change in LMIC compares to United States norms and to assess the applicability of existing tools for monitoring early newborn weight change, we conducted a secondary analysis of this existing dataset to compare weight trajectories in the first 5 days of life among newborns born in our LMIC cohort to weight trajectories depicted in the existing NEWT tool.

## Methods

As previously described^7^, between April 2019 and March 2020, a convenience sample of 741 newborns ≥ 2000 g at birth whose mothers were aged ≥ 18 years, intending to breastfeed for at least six months, and willing to provide informed consent, were enrolled at selected health facilities in Guinea-Bissau, Nepal, Pakistan, and Uganda. Consistent with the WHO Multicentre Growth Reference Study (MGRS) used to generate CGS charts, newborns with major congenital anomalies, danger signs, respiratory distress, or maternal or infant contraindications to breastfeeding were excluded from our study; otherwise, maternal health status did not affect eligibility either for our study or for the study population of the NEWT nomograms. In contrast to MGRS, newborns with economic or environmental constraints on growth were not excluded from our study. To improve comparability with the NEWT tool, we included newborns regardless of feeding type who were delivered at hospitals where exclusive breastfeeding was the most common initial feeding method practiced. The NEWT tool was selected for comparison because it is the most widely used newborn weight loss tool; NEWT nomograms depict quantiles of newborn weight change by hour of age, thus facilitating understanding of an individual infant’s newborn change in the context of reference norms.

Trained study staff obtained duplicate birth weights using a standardized protocol for naked newborns within 6 h of birth and at 1, 2, 3, 4 and 5 days with a Seca 334 scale (Seca Inc., Wandsbek, Germany) accurate to ± 5 g; two additional measurements were taken if the initial two measurements varied by 15 g or more. Follow-up weights were measured at the enrollment health facilities or during home visits as preferred by the study participants. Study participants were traced and located using provided contact information and maps as necessary. All enrolled infants received usual care prior to, during, and after study enrollment. No direct care was provided by the study team, and ill infants were referred. Travel reimbursement was provided; no other incentives were provided.

To generate nomograms for our cohort, we used quantile regression methods appropriate for longitudinal data to estimate the 25th, 50th (median), and 75th percentiles of weight change as a function of time after birth, separately for each country. We applied the penalized fixed-effects model in the R package “Regression Quantiles for Panel Data (rqpd)” to estimate the percentile curves^8^. The model is an extension of ordinary quantile regression methods to longitudinal settings and includes separate intercept terms for each infant, with regularization used to estimate the intercepts. The amount of regularization was controlled by a tuning parameter set to 5.

We used a natural spline with 4 degrees of freedom to estimate non-linear quantile curves as a function of time. One complicating feature of the data is that the first weight (birth weight) was recorded up to 6 h after birth. Based on our previous work, newborns typically lose weight in the first 6 h after birth, so the weight recorded may have been an underestimate of birth weight^6^. Therefore, we imputed birth weights for all newborns based on the NEWT curves prior to fitting the models; specifically, we randomly selected a percentile value to impute at 6 h from the NEWT curves. We then used linear interpolation to impute weight at time 0 based on the time from birth that the weight was recorded and the randomly selected percentile value. For example, with a first weight of 3000 g recorded at 3 h, linear interpolation based on the 95th percentile of the NEWT curves (− 0.236% at 6 h) and the 5th percentile (− 1.94% at 6 h) results in birth weights of 3004 g and 3029 g, respectively. The main assumption with our imputation approach is that the percentile estimates from our previous work reasonably approximate the weight loss in these cohorts for the first 6 h. While the randomly selected percentile is not necessarily accurate for individual newborns, we expect errors to average out across all newborns. The resulting nomograms were then superimposed on existing NEWT nomograms to allow visual comparison, since no statistical test exists to determine whether one nomogram differs from another nomogram^9^.

As a sensitivity analysis, rather than random assignment, we also imputed birth weights based on selecting the 5th percentile for all newborns and separately the 95th percentile for all newborns. The results were similar, likely because the models are primarily based on weight values at later time points and most birth weights were recorded within 4 h of birth.

This study was approved by the UCSF Institutional Review Board, the Guinea-Bissau National Committee on Ethics in Health (Comite Nacional de Etica na Saude), the Nepal Health Research Council, the Institutional Review Committee of Kathmandu University Teaching Hospital, the Ethical Review Committee at the Aga Khan University in Pakistan, the Higher Degrees, Research and Ethics Committee of Makerere University, and the Uganda National Council of Science and Technology. Informed consent for study participation was obtained from all participating mothers. Informed consent for all children was obtained from the mother of each child. All study activities were performed in accordance with relevant guidelines and regulations.

## Results

Of 741 enrolled newborns, 631 were delivered at hospitals where exclusive breastfeeding was the most common initial feeding method practiced and were included in this secondary analysis. Of these 631 newborns, 612 (97%) had weights recorded on the day of birth within 6 h after birth and were included in the subsequent analyses. Among these 612 included newborns, measurement of weight at 1, 2, 3, 4, and 5 days of age were available for 604 (99%), 598 (98%), 594 (97%), 592 (97%), and 587 (96%) newborns, respectively. In Guinea-Bissau, Nepal, Pakistan, and Uganda, weight was first measured at a median [interquartile range (IQR)] of 2 (0.9 to 3.4), 3.7 (0.2 to 5.3), 0.9 (0.2 to 2.0) and 3.9 (2.8 to 4.8) hours, respectively, and mean birth weight [standard deviation (SD)] was 3092 ± 436, 2905 ± 404, 2896 ± 402, and 3138 ± 406 g, respectively. In this cohort, 36 (5.9%) infants were documented to have lost > 10% of their birth weight. Among 476 (78.7%) newborns with reported gestational age, gestational age was 38.3 ± 1.0 (range 36–41) weeks.

**Figure 1:**
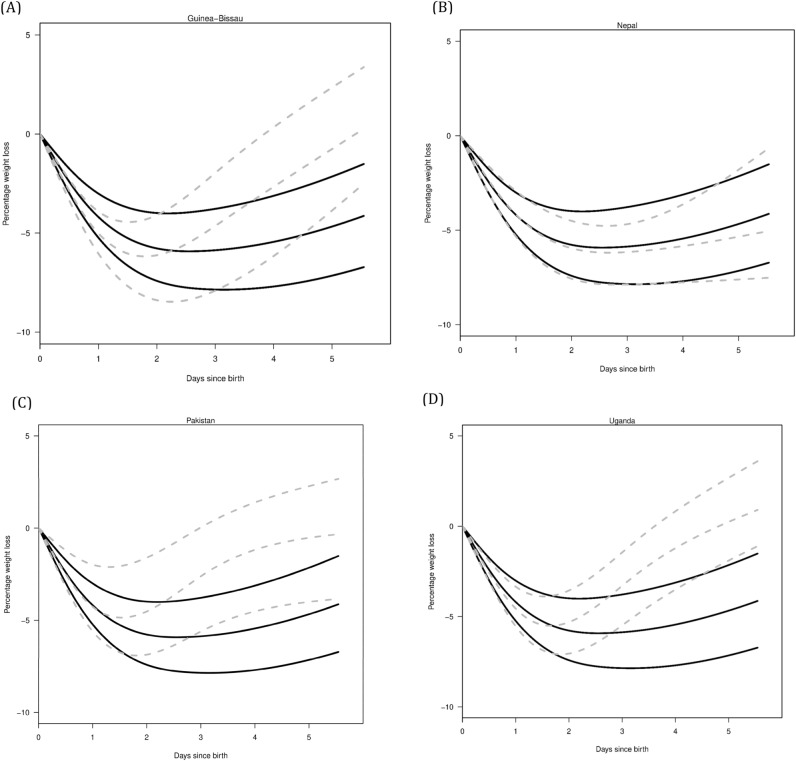
Percentiles (25th, 50th, and 75th) of percentage weight change in newborns during the first 5 days after birth (dotted gray lines) by country [(**A**) Guinea-Bissau; (**B**) Nepal; (**C**) Pakistan; (**D**) Uganda] with NEWT percentiles (black lines) also shown.

The trajectories of initial newborn weight loss prior to nadir in Nepal, Pakistan, and Uganda were similar to United States norms, and in Guinea-Bissau was greater than United States norms (Fig. 1). In Nepal, the subsequent trajectory of newborn weight gain following nadir was similar to United States norms but in Guinea-Bissau, Pakistan, and Uganda, the subsequent trajectories were more pronounced than United States norms.

## Discussion

A graphical depiction of early trajectories of weight change demonstrates that newborns in LMIC have a trajectory of initial weight loss prior to weight gain that is similar to United States’ norms, followed by a trajectory of weight gain faster than US norms. Existing HIC tools for assessing newborn weight loss after birth may be reasonable for use in LMIC, while tracking subsequent weight gain may require the development of new tools. The use of existing HIC tools to monitor the initial period of weight loss may allow LMIC providers to track newborn weight over the first few days to ensure prompt follow-up for those with more pronounced weight loss (e.g., trajectory below the 25th centile). Since newborn weight decreases rapidly in the first day after birth, the use of existing HIC tools might also be a useful adjunct to care or for scientific investigation in LMIC when birth weight is not immediately obtained, by allowing extrapolation of birth weight from weights obtained up to 3 days after birth.

Since newborn weight may potentially be measured at varying hours and potentially, days of age in LMIC, we believe such weight loss tools could be of beneficial clinical utility to healthcare providers in LMIC as they are in HIC, informing and helping to calibrate clinical management decisions, including discharge planning, need for lactation and feeding support, and timing and type of newborn follow-up^3,10,11^. Thus, while newborn populations are different in LMIC settings, these data demonstrate the consistency and robustness of existing tools to track newborn weight loss worldwide. The fact that the trajectory of weight gain following nadir appears to be faster in LMIC than in HIC might potentially be attributable to intrauterine growth restriction in the LMIC cohort followed by compensatory weight gain after birth. If this is the case, further research to identify the optimal trajectory of initial weight gain in LMIC would be important.

Our study had several important limitations. Our convenience sampling strategy at selected health facilities in four LMIC may limit the generalizability of our results to newborns in any given country or region or worldwide. However, the similarity of initial newborn weight change patterns in our LMIC cohort to initial newborn weight change patterns in the United States suggests that existing HIC tools may be useful globally. The NEWT tool was developed from a newborn cohort that included some late preterm newborns but excluded those < 36 weeks gestational age, while our cohort did not have reliable data on gestational age for all newborns. Nevertheless, both studies included only newborns born ≥ 2000 g, and given that no newborns with recorded gestational age had a gestational age of < 36 weeks, it is unlikely that our study enrolled any very or extremely preterm newborns. Few infants in our cohort were documented to have lost > 10% birth weight, so we did not have adequate sample size to determine the predictive value of the nomograms for predicting weight loss > 10% birth weight. Although we could not demonstrate predictive validity for this outcome, our results do provide a method for identifying LMIC newborns whose weight loss is more pronounced than the 25th, 50th and 75th centiles, which might potentially allow for closer follow-up and improved outcomes for newborns with more pronounced weight loss.

Overall, our study results demonstrate that initial newborn weight loss at birthing facilities in four LMIC countries is similar to that demonstrated using existing HIC tools for tracking newborn weight loss. Clinicians in LMIC may consider using these existing tools to follow infants clinically in the first 48 h after birth, and investigators may consider using these tools to account methodologically for variation in the timing of measurement of birth weight when not obtained immediately following birth. Our study cohort included late preterm infants and those with birth weights ≥ 2000 g, and thus our findings may be applicable to many late preterm and low birth weight infants, who are disproportionately located in LMICs^12^. Since initial monitoring of newborn weight loss is standard-of-care in HIC settings, these nomograms have the potential for wide clinical and scientific applicability in LMIC for early identification of newborns on a trajectory for greater weight loss and related morbidities.

## Data Availability

De-identified, individual-level data on variables presented in this paper will be shared with other researchers after contact of the corresponding author, approval of the proposed research question(s) and a signed data use agreement.
